# Letter to the Editor on the systematic review “Narrow band imaging versus white light cystoscopy alone for transurethral resection of non-muscle invasive bladder cancer”

**DOI:** 10.1007/s00345-023-04435-7

**Published:** 2023-06-13

**Authors:** Morgan Roupret, Max Burger, Arnulf Stenzl

**Affiliations:** 1 Sorbonne University, GRC 5 Predictive Onco-Uro, AP-HP, Urology, Pitie-Salpetriere Hospital, F-75013, Paris, France; 2 Department of Urology, Caritas St. Josef Medical Center, University of Regensburg, Regensburg, Germany; 3 Department of Urology, Eberhard Karls University of Tübingen, Tübingen, Germany

Dear editors,

Recently, two Cochrane reviews on narrow band imaging (NBI) [1] and blue light cystoscopy (BLC) [2] were published.

While our disclosures of clinical and academic interest, lecturing and advising must be taken into account, we are convinced that the quality of TURBT needs improvement.

Both Cochrane reviews use the same methods showing reduced risk of recurrence by 37% and 34% for NBI and BLC and of progression by 35% for BLC; no progression data were available on NBI.

We esteem Cochrane and acknowledge the reviews; there is the impression of a less rigorous interpretation and less balanced discussion of the NBI compared to the BLC data.

## Selection of NBI studies

Six RCTs were included in the NBI review [1]. Three trials [3–5] failed to meet primary endpoints. Also, non-standard techniques were used, e.g., plasma-vaporization [6, 7] biasing results [8–10]; *p* values are missing; such is unexpected (Table 1).

**Table 1:** Included publications in the NBI Cochrane report with results of the recurrence endpoint in comparison between NBI and WLC

References	Sample size	Follow-up time	Recurrence rates WL vs NBI	*p* value
Kim 2018 [4]	198	12 months	26% vs 13%	*p* = 0.3
Lee 2014 [3]	68	16 months (median)	73% vs 77% recurrence-free survival	*p* = 0.416
Ma 2015 [6]	178	3 and 12 months	38% vs. 19%	*p* < 0.05*
Naito 2016 [5]	965	3 and 12 months	25% vs 27%	*p* = 0.585
Naselli 2012 [13]	148	12 months	51.4% vs. 31.6%	*p* = 0.014*
Stănsecu 2014 [7]	260	12 and 24 months	26% vs. 12%	No *p* value reported

*Sig *p* < 0.05

## Discussion of validity of studies

In the BLC report [2], missing blinding is emphasized as limitation; in contrast, the NBI report [1] describes such as “the nature of TURBT”.

## Discussion of validity of effects

The BLC report [2] emphasizes the BLC effect being dependent on baseline risk; no such conclusion is found in the NBI report [1]. BLC and NBI are similarly classified as having low levels of certainty. Surprisingly, the NBI compares NBI with BLC despite lacking comparative data and does not even mention the considerably larger numbers of RCTs and sample sizes for BLC including progression [11, 12].

## Discussion of clinical application

Despite being out of scope, the NBI report [1] emphasizes the ease of use of NBI vs. BLC; this report is not a systematic comparison between BLC and NBI and both are used with ease.

## Discussion of clinical impact

The pivotal event for NMIBC is progression; the NBI report [1] does not even mention respective data lacking for NBI, while it compares NBI with BLC despite comparative data.

## Overall impression

In conclusion, considering the high-quality standards of the Cochrane reviews, differences in rigor and criticism of the evaluation and interpretation of BLC and NBI are tangible. We suggest the said aspects to be considered.

## Our view on the integration of BLC into current management of MIBC

The Cochrane review showed that BLC-guided TURB may reduce recurrence rates; such improvement is needed [14]. Interpreting data on BLC, lower number of recurrences than statistically assumed must be considered. Most patients were at low and intermediate risk, which is no longer the appropriate target population recommended by the EAU guidelines, i.e., high risk.

BLC may guide management following TURBT [14]; adjuvant therapy is most important for prognosis of NMIBC. Many devices in urology have a greater benefit than demonstrated in studies due to confounders, such as adjuvant therapy. In general, we all recognize that better visualization of the bladder is improving the quality of TURBT. Even basic cystoscopy has evolved without being validated by RCTs. Thus overall, we do consider BLC a valuable tool available in the urologist’s armamentarium.
